# Isolation and preliminary pathogenicity of a recent feline astrovirus strain in China

**DOI:** 10.3389/fvets.2025.1614686

**Published:** 2025-07-22

**Authors:** Wenjie Wang, Ya Zhang, Xiang Wang, Xu Zhu, Li Gong, Zheng Jing, Ting Meng, Jiayu Shen, Yeping Tan, Tong Qin, Zhimin Li, Zhenwei Bi

**Affiliations:** ^1^School of Pet Science and Technology, Jiangsu Agri-Animal Husbandry Vocational College, Taizhou, China; ^2^Institute of Veterinary Medicine, Jiangsu Academy of Agricultural Sciences, Key Laboratory of Veterinary Biological Engineering and Technology, Ministry of Agriculture and Rural Affairs, National Center for Engineering Research of Veterinary Bio-products, Nanjing, China; ^3^GuoTai (Taizhou) Center of Technology Innovation for Veterinary Biologicals, Taizhou, China; ^4^College of Animal Science and Technology, Hebei North University, Zhangjiakou, China; ^5^Shanghai GlinX Biotechnology Company Limited, Shanghai, China; ^6^Institute of Animal Sciences of Chinese Academy of Agricultural Sciences (CAAS), Beijing, China

**Keywords:** FeAstV, isolation, pathogenicity, diarrhea, co-infection

## Abstract

Feline astroviruses (FeAstVs) have been increasingly detected in cats in recent years, yet their isolation and pathogenicity remain poorly characterized. In this study, we screened 86 feline diarrheal samples and identified FeAstV in 9.3% (8/86) of the cases, most of which were co-infected with feline parvovirus (FPV). A FeAstV strain (22SDWH1003-16) was successfully isolated in F81 cells from a single FeAstV positive sample, inducing cytopathic effects (CPEs) over 15 passages. The phylogenetic tree of ORF2 classified the isolate within *Mamastrovirus 2* group 1, the most common in the inter-specific transmission within cats. Experimental inoculation of four cats revealed seroconversion in all animals, transient fecal shedding in 3/4 cats, and self-limiting diarrhea in one individual. Co-infection experiments demonstrated enhanced FeAstV replication in the presence of FPV. Our findings provide the direct evidence of FeAstV-induced diarrhea in cats and highlight the role of viral co-infections in disease severity.

## 1 Introduction

Astroviruses (AstVs), members of the family *Astroviridae*, are non-enveloped spherical virions characterized by distinctive star-shaped surface structures observable through electron microscopy. The AstV genome consists of a small single-stranded RNA molecule (~6–7 kb) containing three overlapping open reading frames (ORFs): *ORF1a, ORF1b, and ORF2*. According to the International Committee on Taxonomy of Viruses (ICTV),^1^ the family *Astroviridae* is officially divided into two genera based on ORF2-encoded capsid protein analysis: Mamastrovirus (infecting mammalian species) and Avastrovirus (infecting avian species). With the discovery of diverse AstV genotypes in animal species, the number of Mamastrovirus species has increased from 19 to 33, and Avastrovirus species have recognized 7 species from 3 since 2019 (1). These findings suggest that the known host range of the family *Astroviridae* infections is continuously updated.

Human astroviruses (HAstVs) are well-eatablished etiological agents of pediatric gastroenteritis (2–6). Similarly, AstV infections in other most species have been epidemiologically associated with gastroenteritis. Notably, extraintestinal pathologies have been documented across multiple hosts: AstVs are linked to interstitial nephritis in young chickens (7), fatal hepatitis in ducks (8), and neurological diseases in swine (9). Feline astrovirus (FeAstV) was first identified in diarrheic domestic kittens by electron microscopy (EM) in the 1980s (10). Following its initial characterization, FeAstVs have achieved worldwide prevalence (11–14). Recent years, increasing numbers of reports showed frequent detection of FeAstVs in both asymptomatic and diarrheic cats, with significantly higher prevalence in clinical cases in China (14). Epidemiological data reveal significantly higher FeAstV prevalence in diarrheic cats compared to asymptomatic individuals (15), suggesting a potential association with gastroenteritis. However, this correlation remains presumptive due to critical evidentiary gaps: the absence of *in vitro* isolates precludes fulfillment of Koch’s postulates, and frequent co-detection with enteric pathogens (e.g., feline coronaviruses, parvoviruses) confounds causal attribution.

Here, we reported the isolation of a recent Chinese FeAstV strain from a single FeAstV-infected clinical specimen using F81 cells and carried out virus-challenged experiments to confirm the pathogenic potential of FeAstV in cats. This provides a reference for further understanding of clinical cases of FeAstV infection in cats, and laid the foundation for prevention and control of FeAstV infection.

## 2 Materials and methods

### 2.1 Sample collection, virus detection and cells culture

During August–September 2022, a total of 86 diarrheic fecal specimens were collected from domestic cats using rectal swabs at three veterinary hospitals located in Weihai (Shangdong Province), Hefei (Anhui Province) and Changsha (Hunan Province). These samples were detected and screened for feline parvovirus (FPV), feline coronavirus (FCoV), feline bocavirus (FBoV), feline astrovirus (FeAstV) and feline chaphamaparvovirus (FChPV) using a feline gastrointestinal tract five joint kits (InCycle, GlinX Company, China) following the manufacturer’s protocol. For cell culture, F81 cells were propagated in complete growth medium consisting of DMEM (Gibco, USA) supplemented with 10% heat-inactivated fetal bovine serum (FBS; Gibco, USA), 100 U/ml penicillin, and 100 μg/ml streptomycin sulfate, maintained at 37°C in a humidified 5% CO2 atmosphere.

### 2.2 Viral isolation and titer determination

The rectal swab sample was collected in 1 ml PBS containing penicillin streptomycin antibiotic in a 1.5 ml EP tube. Samples were vortexed vigorously for 30 s, then centrifuged at 12,000 × *g* for 5 min at 4°C to remove particulate matter. The supernatant was filtered through a 0.22 μm pore-size membrane (Millipore) to eliminate bacterial contamination. Filtered supernatants were immediately aliquoted and stored at −80°C until inoculation. For viral isolation, 200 μl of processed supernatant was inoculated onto confluent F81 cell monolayers in 6-well plates, adsorbed for 2 h at 37°C with 5% CO₂, then discarded and maintained in DMEM supplemented with 2% FBS. Parallel negative controls were maintained using sterile phosphate-buffered saline. Cellular morphology was monitored at 24-h intervals for cytopathic effect (CPE) assessment. Upon observation of characteristic CPE, 15 successive blind passages were performed to confirm viral stability. Viral titers were determined as 50% tissue culture infectious dose (TCID_50_/ml) using the Reed-Muench endpoint dilution assay with four replicates per dilution.

### 2.3 Detection and amplification of FeAstV *ORF2* gene

Total RNA was extracted from FeAstV-infected cells using E.Z.N.A. Viral RNA Kit (Solarbio, China) following the manufacturer’s protocol. Subsequently, 1 μg of RNA were reverse-transcribed into cDNA with the Hifair® AdvanceFast 1st Strand cDNA Synthesis Kit (Yeasen, China). For FeAstV detection, RT-PCR targeting a 418-bp conserved region of the *ORF1b* gene was performed using specific primers (forward: 5’-GAAATGGATTGGACACGYTAYGA-3′; reverse: 5’-GGCTTGACCCACATRCCGAA-3′) under the following conditions: 94°C for 10 min initial denaturation, 35 cycles of 94°C for 1 min, 55°C for 30 s and 72°C for 1 min; followed by a final extension at 72°C for 10 min. To analyze genetic characteristics, the complete *ORF2* gene was amplified using primers (forward: 5′-ATGGCTAGC AAGYCTGGYAAAGAAG-3′; reverse: 5′-GCGTGGCCTCGGCT CTCAA-3′) (15) with modified cycling parameters: 94°C for 5 min, 35 cycles of 94°C for 1 min, 65°C for 1 min and 72°C for 2 min; and a final extension at 72°C for 10 min. The PCR products were analyzed by 1% agarose gel electrophoresis and the 2,438-bp ORF2 fragment was sequenced by Tsingke Biotech (Beijing, China).

### 2.4 Phylogenetic analyses

Full-length ORF2 nucleotide sequences of FeAstV strains were obtained from GenBank and aligned with the isolate from this study using ClustalW in MEGA 11. A phylogenetic tree was constructed using the neighbor-joining method with 1,000 bootstrap replicates. Variability of amino acid sequence within the complete *ORF2* gene was analyzed through Jotun Hein method in DNASTAR software.

### 2.5 Animal challenge experiments

Six experimental cats (breed: Chinese native cat; age: 6–8 weeks; mean weight: 1,000 ± 100 g; sex: 3 male/3 female) were purchased from a pet market in Nantong City, Jiangsu province of China. All cats underwent 14-day acclimatization with daily health monitoring (temperature, appetite, activity) prior to inoculation. Fecal sample from all cats tested negative for FeAstV, FPV, FCoV, FBoV-1, FeChPV using five joint kits (InCycle, GlinX Company, China). Serum samples were additionally confirmed negative for FeAstV antibodies. The cats were randomly assigned to either a control group (*n* = 2) or a FeAstV-challenged group (*n* = 4), with all animals individually housed in separate isolation units. Both groups exhibited normal fecal consistency and showed no clinical signs prior to inoculation. The challenge group received 2 ml (10^3^ TCID_50_/ml) of isolated FeAstV strain suspension via subcutaneous injection combined with 8 ml oral administration, while control animals were administered equivalent volumes of phosphate-buffered saline (PBS) though identical routes. Through the study, the two groups were maintained in physically separated rooms under continuous observation. Clinical symptoms were monitored and recorded daily with rectal temperature measurements and anal swabs collections performed every 48 h for FeAstV detection via ORF1b-targeted RT-PCR.

### 2.6 Immunoperoxidase monolayer assay (IPMA)

Serum samples from FeAstV-challenged cats (experimental group) and uninfected controls were analyzed for FeAstV-specific antibodies via IPMA. Briefly, F81 cells cultured in 24-well microplates were infected with 22SDWH1003-16-FeAstV strain for 72 h, with uninfected F81 cells serving as negative controls. Following infection, cells were fixed with 4% paraformaldehyde at 4°C for 30 min and blocked with PBST containing 10% FBS. Diluted serum samples (1: 200 in PBS) from both groups were independently applied to the monolayers and incubated at 37°C for 1 h. After three PBST washes, cells were incubated with HRP-conjugated goat anti-cat IgG (1: 1000) for 1 h at 37°C. Following additional washes, chromogenic development was performed using ACE peroxidase substrate (Biodragon, China) at 37°C for 5 min. Immunoreactivity was visualized and documented using an inverted microscope (Olympus, Japan).

### 2.7 FeAstV and FPV co-infection assay

F81 cells were grown to 80 to 90% confluence in 12-well plates, then were co-inoculated with FeAstV and FPV for 96 h, with monoinfection groups (FeAstV-only and FPV-only) serving as controls. Viral replication of the two viruses in F81 cells were assessed by real-time PCR through the SYBR Green Master (Yeasen) with specific primers as following (16, 17): FPV forward: 5′-CTGGAGGACGAGGGATACAGTGAC-3′, FPV reverse: 5′-GGT CGCCGAGGAGGACAAGG-3′ FeAstV forward: 5′-GAGAAGT ATGCAGGGGTCCA-3′ FeAstV reverse: 5′-CAAAGGCTTGTAG CCAGAGGT-3′ β-actin forward: 5′-GACTACCTCATGAAGATC CTCACG-3′ β-actin reverse: 5′-CCTTGATGTCACGCACAATT TCC-3′, qPCR was performed on StepOnePlus Real-Time PCR System and the relative mRNA levels were calculated by the 2^-ΔΔCT^ method, including normalization to CT values of β-actin.

### 2.8 Statistical analysis

Data are expressed as the mean±standard deviation (SD). Statistical significance was performed using GraphPad Prism 8 (GraphPad Software, USA). The student’s t-test was employed to determine statistical differences between the two groups. In the figures, asterisks indicate statistical significance, with * representing *p* < 0.05.

## 3 Results

### 3.1 Prevalence of FeAstV in feline diarrhea samples

During 2022, screening of 86 diarrheic feline fecal samples from three Chinese pet hospitals (Weihai, Hefei and Changsha) using feline gastrointestinal tract five-joint kits (Shanghai GlinX Company) revealed predominant infections by FPV (48.84% [42/86]) and FCoV (48.84% [42/86]), followed by FeAstV (9.30% [8/86]), FBoV (2.33[2/86]), and no FChPV detection (Table 1). Coinfection analysis demonstrated 16.28% (14/86) FcoV/FPV, 8.14% (7/86) FPV/FeAstV, 2.32% (2/86) FcoV/FBoV-1 and 2.32% (2/86) triple FCoV/FPV/FBoV-1 infections, with 54.76% (23/42) of FPV-positive and 42.86% (18/42) of FCoV-positive samples showing concurrent infections (Table 2).

**Table 1:** Detection rate of five viruses in clinical diarrhea samples from cats.

Types of viruses	FCoV	FPV	FeAstV	FBoV-1	FeChPV
Positive statistics	42/86	42/86	8/86	2/86	0/86
Positive rate (%)	48.84	48.84	9.30	2.33	0

**Table 2:** Mixed infections of five viruses in clinical diarrhea samples from cats.

Mixed viral infections	FCoV + FPV	FCoV + FBoV-1	FPV + FeAstV	FCoV + FPV + FBoV-1
Positive statistics	14/86	2/86	7/86	2/86
Positive rate (%)	16.28	2.32	8.14	2.32

Notably, FeAstV exhibited exclusive epidemiological coupling with FPV, where 87.5% (7/8) of FeAstV-positive samples were coinfected with FPV compared to only 12.5% (1/8) monoinfections, suggesting potential viral synergy in pathogenesis. Collectively, these results showed that coinfection should be fully considered in the clinical diagnosis and treatment of diarrhea diseases of cats.

### 3.2 Isolation of FeAstV in F81 cells

**Figure 1:**
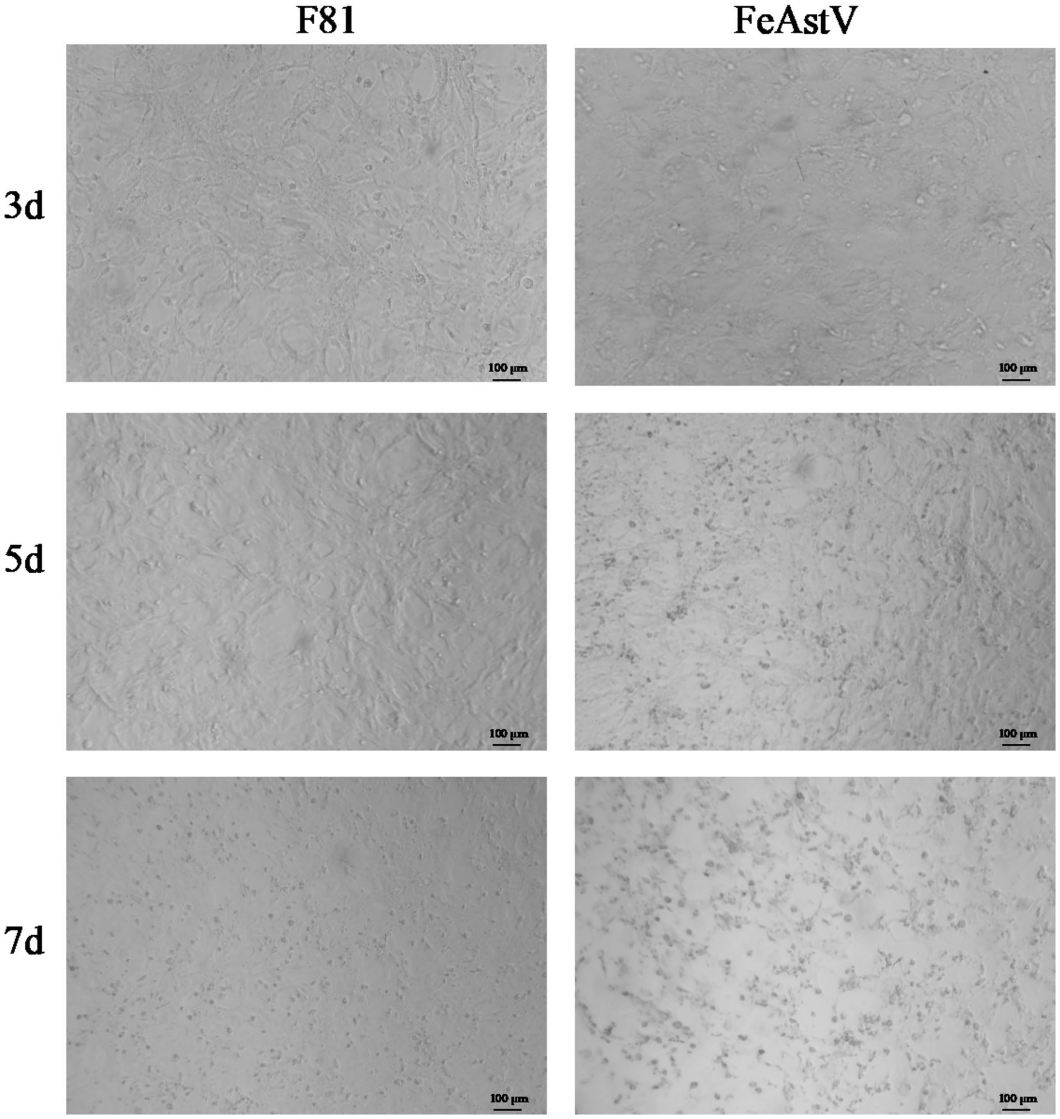
Isolation of FeAstV using F81 cells. F81 cells infected with FeAstV exhibited cytopathic effects (CPE) progressing over time, with initial manifestations observed at 3 days post-inoculation that progressively worsened by days 5 and 7. Unchallenged F81 cell cultures served as normal controls.

**Figure 2:**
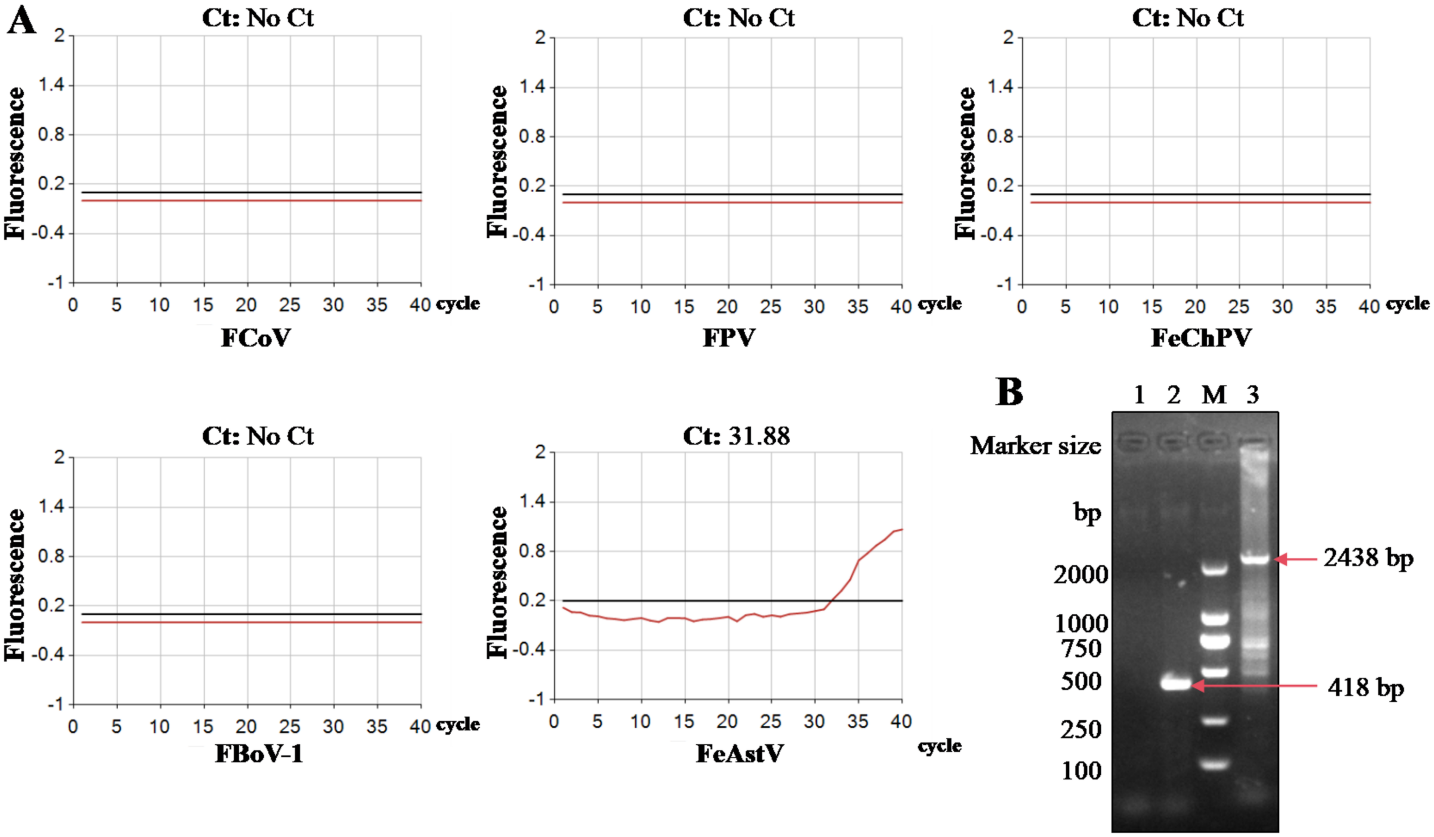
Identification of cell-isolated FeAstV. **(A)** Viral identification was performed using a commercial fluorescent quantitative PCR kit targeting FeAstV and other feline enteric pathogens (FPV, FCoV, FBoV, FChPV); **(B)** The partial ORFb1 and complete ORF2 genes were amplified from cell-isolated FeAstV by RT-PCR. M: DNA marker; 1: normal cells control (partial ORFb1); 2: cell-isolated FeAstV (partial ORFb1); 3: cell-isolated FeAstV (complete ORF2).

We conducted isolation attempts of FeAstV in F81 cell culture using the clinical specimens confirmed to contain exclusive FeAstV infection. Following filtration through a 0.22 μm membrane, one FeAstV-positive sample was incubated onto F81 cell monolayers. Distinct cytopathic effects (CPE) emerged progressively: initial cell rounding and shrinkage become apparent by day 3 post-inoculation, progressing to pronounced balloon-like cell clustering by day 5. This contrasted with control cells maintaining typical epithelioid morphology. By day 7, extensive cell detachment was observed in infected cultures, while control cells exhibited minimal age-related morphological changes attributable to prolonged culture duration (Figure 1). The viral isolate underwent 15 serial blind passages, with virus harvested on day 5 post-inoculation upon observation of consistent CPE in each passage, confirming successful viral adaptation. To exclude potential co-isolation of other enteric pathogens. The isolate was screened using a commercial feline gastrointestinal tract five-joint kits (FPV, FeAstV, FBoV-1, FCoV, FeChPV) (Figure 2A) and supplementary (RT)-PCR assays for by FBuV, FSCV, FCV, FKoV, FNoV, FRV (18). Exclusive FeAstV positivity was confirmed through these comprehensive tests. RT-PCR was also used to identify the isolated FeAstV stain by two sets primers, the specific band was observed in gel (Figure 2B). The *ORF2* gene were amplificated by RT-PCR and sequenced by Tsingke Biotech. The isolate, designated 22SDWH1003-16-FeAstV based on spatiotemporal collection data, demonstrated a tissue culture infectious dose (TCID_50_) of 10^3^/ml. This successful isolation and characterization establish a foundation for subsequent virological investigations of FeAstV pathogenesis and host interactions.

### 3.3 Phylogenic analysis of FeAstV

**Figure 3:**
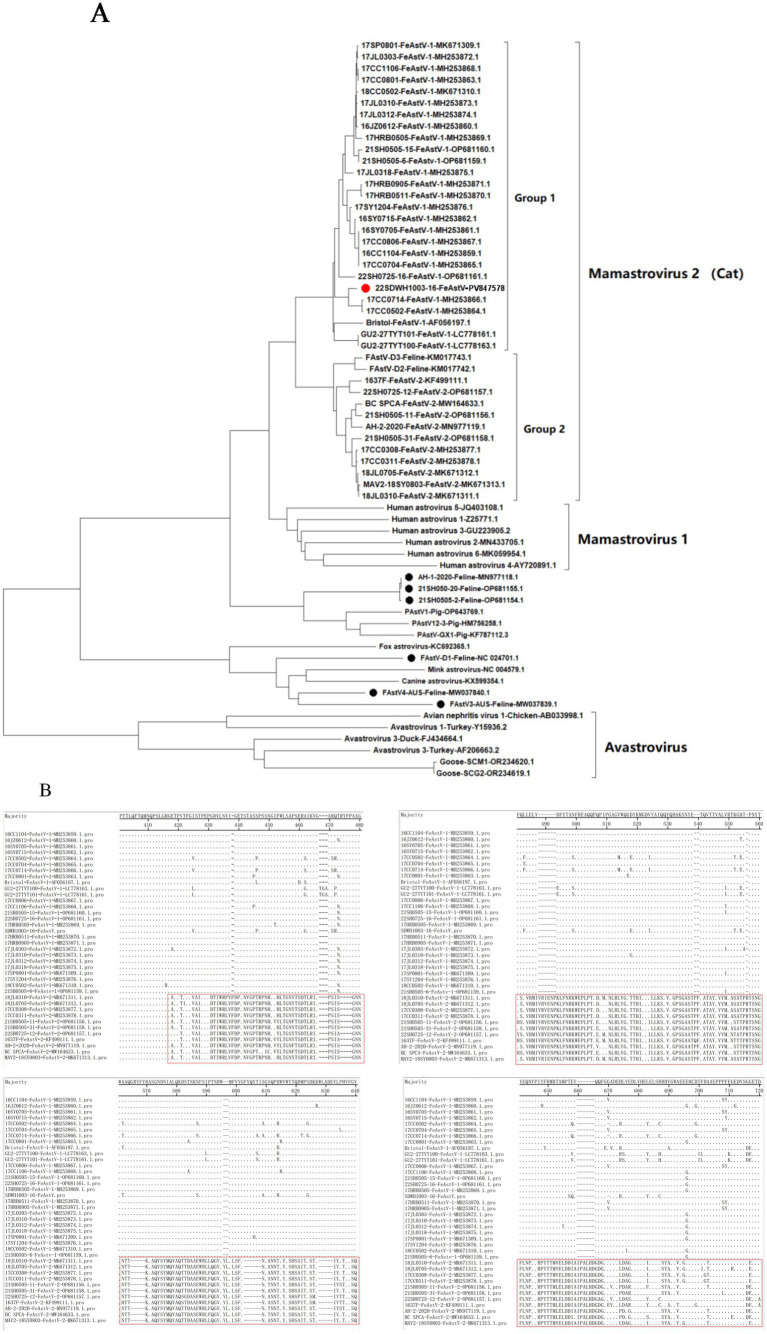
Phylogenetic and sequence analysis of FeAstV strains based on ORF2 gene. **(A)** Neighbor-joining phylogenetic tree constructed with MEGA 11 (1,000 bootstrap replicates). The red circle denotes the FeAstV strain isolated in this study, while black circles represent feline-derived FeAstV strains phylogenetically linked to other mammalian astroviruses. **(B)** Comparative alignment of deduced amino acid sequences of FeAstVs between Group 1 and Group 2 clusters.

As of October 2024, 48 complete ORF2 (capsid) sequences of FeAstV from global isolates including China (*n* = 39), Canada (1), Japan (2), Australia (2), America (3), Netherland (1) were registered in GenBank. A phylogenetic reconstruction based on these reference sequences and the isolated 22SDWH1003-16-FeAstV strain (GenBank accession number: PV847578) revealed distinct clustering within the *Mamastrovirus 2* species, subdivided into two major groups (Group 1 and Group 2). Notably, Group 1 contained a higher proportion of FeAstV sequences globally compared to Group 2, suggesting its potential dominance in viral circulation, particularly within China (Figure 3A). The 22SDWH1003-16-FeAstV displayed highest nucleotide sequence identity (94.2–99.4%) to two FeAstV strains (17CC0502-FeAstV-1-MH253864.1 and 17CC0714-FeAstV-1-MH253866.1) from Sichuan, China in 2017. All three strains clustered within Group 1 of *Mamastrovirus 2*, further supporting its predominance in China (Figure 3A). Comparative analysis of deduced ORF2 amino acid sequences identified significant divergence (positions 418–718) between Group 1 and Group 2 of *Mamastrovirus* 2 (Cat), highlighting structural variations in the capsid protein (Figure 3B). Intriguingly, FeAstV strains from cats in the United States and Australia displayed closer evolutionary ties to astroviruses of mink, canine, and fox origin, corroborating prior findings of cross-species astrovirus relationships (19). Additionally, two Chinese FeAstV strains (21SH050-20 and 21SH0505-2, Shanghai) and the recombinant FeAstV AH-1-2020 (Anhui), previously reported to share ORF2 recombination with porcine astrovirus (PAstV) type 1, formed a distinct clade with PAstV in the phylogenetic tree (Figure 3A). These observations underscore the genetic complexity of FeAstV lineages circulating in China and globally, emphasizing the need for continued surveillance to elucidate recombination dynamics and host adaptation mechanisms.

### 3.4 Infection and pathogenicity of FeAstV

**Figure 4:**
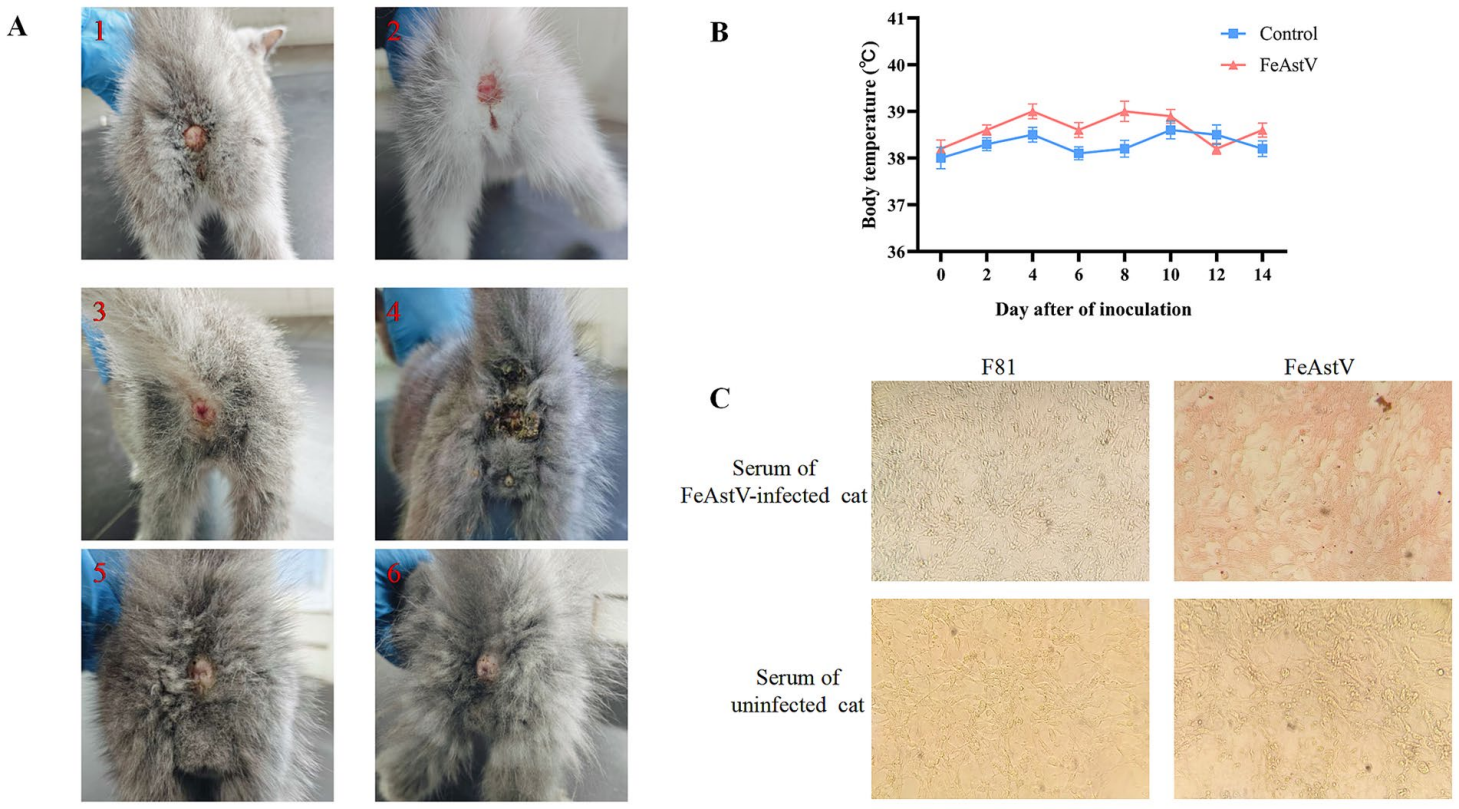
Experimental infection and pathogenicity assessment of FeAstV in felines **(A)** Clinical manifestation of post-inoculation diarrheal symptoms. Gross pathological changes in the perianal region of representative FeAstV-infected subjects (1–4) versus negative controls (5, 6). **(B)** Longitudinal monitoring of core body temperature. Rectal temperatures were recorded at 48-h intervals over a 14-day observation period post-inoculation. **(C)** Humoral immune response profiling by immunoperoxidase monolayer assay (IPMA). Specific reactivity was observed between serum from infected cats and FeAstV-infected F81 cell monolayers, with seronegative controls (naive serum+infected cells; infected serum+uninfected cells) demonstrating assay specificity.

To evaluate the pathogenic potential of FeAstV, six cats were chosen from a pet market, they were negative for antigen and antibodies of FeAstV with confirmed absence of coinfections with common feline viruses (FPV, FcoV, FBoV-1, FeChPV).·The 4 cats of them were randomly chosen as viral challenge group, while two served as controls, maintained in separate isolation facilities. Challenged cats received combined subcutaneous and oral inoculations of FeAstV (22SDWH1003-16-FeAstV), with daily clinical monitoring and biennial anal swab collection for RT-PCR detection over a 22-day observation period. Clinical outcomes revealed transient diarrheal symptoms in 1/4 challenged cats (days 2, 4, 6, and 8 post-inoculation), resolving completely by day 10. Asymptomatic infections were observed in both the remaining challenged cats and controls (Figure 4A; Table 3). Viral RNA shedding was detected in 3/4 challenged cats: the symptomatic individual tested positive on day 6, while two asymptomatic cats shed virus on days 2 and 3 (Table 3). Serum samples collected 14 days post-infection confirmed seroconversion in all challenged cats by immunoperoxidase monolayer assay (IPMA), whereas controls remained seronegative (Figure 4C). No significant thermometric differences emerged between groups (Figure 4B). Collectively, FeAstV can infect and cause shedding in cats, but it is not always accompanied by clinical symptoms.

**Table 3:** Clinical symptoms and RT-PCR detection of FeAstV in rectal swabs from infected cats.

Day		0	2	4	6	8	10	12	14	16	18	20	22	Groups
Clinical symptoms	1	N	N	N	N	N	N	N	N	N	N	N	N	Experimental group
	2	N	N	N	N	N	N	N	N	N	N	N	N	
	3	N	N	N	N	N	N	N	N	N	N	N	N	
	4	N	D	D	D	D	N	N	N	N	N	N	N	
	5	N	N	N	N	N	N	N	N	N	N	N	N	Control group
	6	N	N	N	N	N	N	N	N	N	N	N	N	
RT-PCR	1	-	-	+	-	-	-	-	-	-	-	-	-	Experimental group
	2	-	-	-	-	-	-	-	-	-	-	-	-	
	3	-	+	-	-	-	-	-	**-**	-	-	-	-	
	4	-	-	-	+	-	-	-	-	-	-	-	-	
	5	-	-	-	-	-	-	-	-	-	-	-	-	Control group
	6	-	-	-	-	-	-	-	-	-	-	-	-	

D: diarrhea, N: no diarrhea; +: FeAstV-positive, −: FeAstV-negetive.

### 3.5 The effect of co-infection of FeAstV-FPV on each other

**Figure 5:**
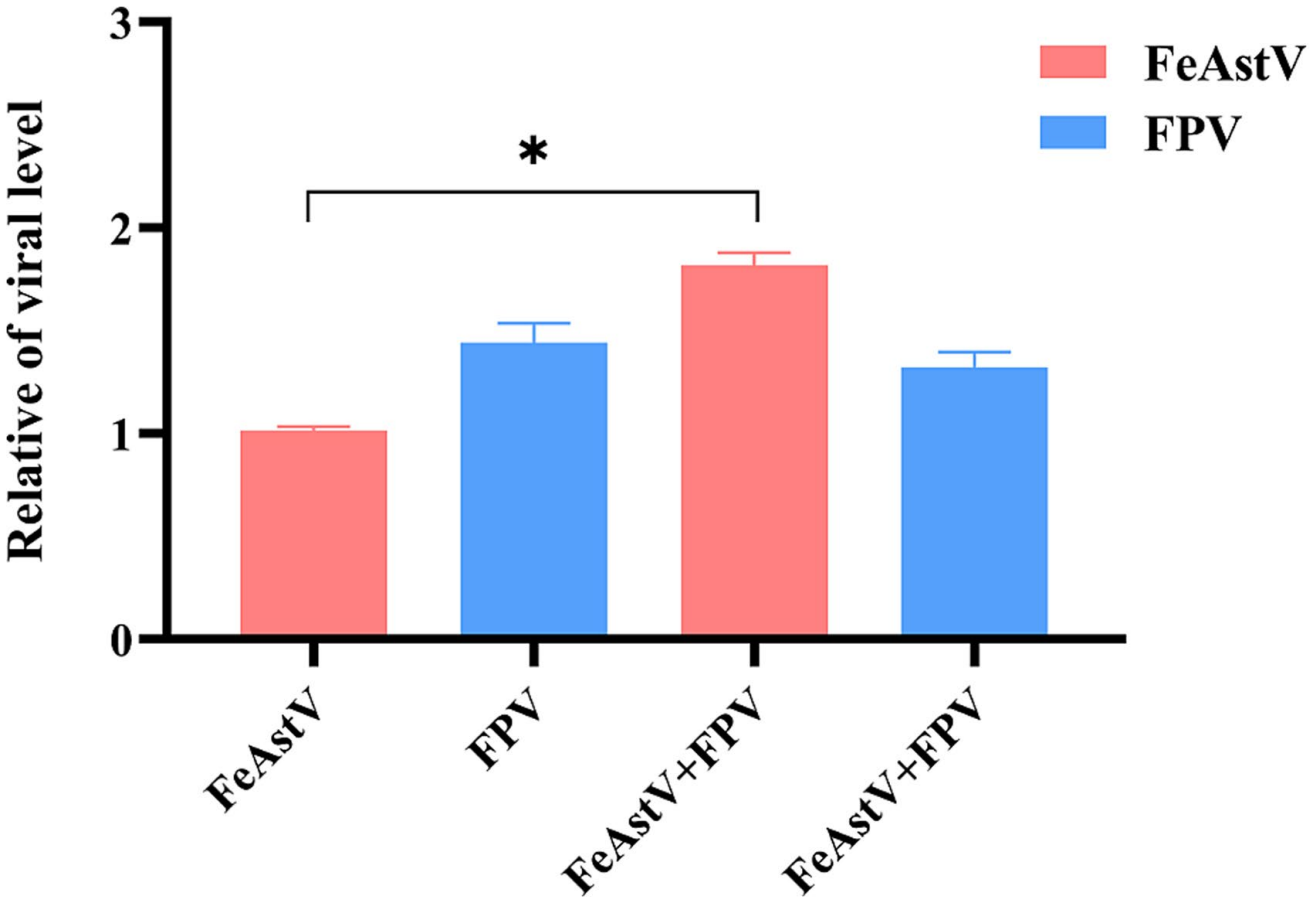
The effect of co-infection of FeAstV and FPV on each other. *In vitro* co-infection modeling in F81 cells with simultaneous inoculation of FeAstV and FPV, followed by quantitative RT-PCR-based monitoring of cross-regulated viral replication.

The prevalent Chinese strain FPV SDYT39 (GenBank accession number: OQ535512) was employed in co-infection experiments with FPV and FeAstV (18). The F81 cells were co-infected with FPV and FeAstV at a multiplicity of infection (MOI) of 0.1, the effect on viral replication between each other was determined on the third day after infection. Compared to FeAstV infection alone, the replication of FeAstV was increased in co-infected cultures of FeAstV and FPV, indicating that FPV promoted the replication of FeAstV when co-infection happened. We did not observe the effect of co-infected FeAstV on replication of FPV (Figure 5). These findings reveal an asymmetric viral interaction where FPV potentiates FeAstV replication without reciprocal inhibition.

## 4 Discussion

Existing epidemiological studies have revealed significant geographical variations in feline astrovirus (FeAstV) detection rates globally. Early investigations in Australia (1984–1985) demonstrated a 4.8% prevalence (11/228), with comparable detection rates between diarrheal (6/62) and asymptomatic cats (5/166) (18). Subsequent surveys in the United States showed marked regional differences, with Florida reporting detection rates of 10% (5/50) and 28.7% (35/122) across two separate studies during 2012–2014 (20). Asian data from South Korea’s Mudan Market Animal Hospital (2011) revealed a 17.7% prevalence (11/62) (21), while Chinese studies have provided more recent epidemiological insights. Notably, the first northeastern China surveillance (2018–2021) identified a substantial 23.4% infection rate (46/197) in domestic cats (15), contrasting with lower detection rates (6.06%, 2/33) observed in Anhui Province (22). Expanded surveillance across multiple provinces (Anhui, Jiangsu, Shanghai, Guangdong) during 2018–2021 revealed concurrent circulation of multiple pathogens: FeAstV (12.59%, 17/135), FPV (48.89%, 66/135), FBoV-1 (20.74%, 28/135), and FeKoV (19.26%, 26/135), with polyviral infections occurring in 25.19% (34/135) of cases (23). Our current investigation identified a 9.3% overall prevalence (8/86), reinforcing the concept of geographical variability in FeAstV distribution. These findings emphasize the importance of region-specific viral surveillance in feline populations, particularly given the frequent viral co-infections documented across studies. The observed 25.19% co-infection rate in our work aligns with previous reports of FeAstV-FPV synergistic infections (24), suggesting this viral combination may represent a common pathogenic consortium in feline enteric disease. This pattern underscores the need for comprehensive diagnostic approaches in clinical settings to address complex infection dynamics.

The *RdRp* region (*ORF1b*) represents the most conserved part of the astroviral genome and often is used as a target for viral detection (25). In contrast, the highly variable ORF2 gene encoding the capsid protein is used for evolutionary analysis, playing a vital role in virus structure organization, antigenicity, host infectivity and cellular trafficking (26). Our phylogenetic analysis of complete ORF2 sequences from GenBank-archived FeAstV strains revealed the large FeAstV strains belonged to *Mamastrovirus* 2 in phylogenetic tree, which segregated into two major subgenotypes (group 1 and group 2), confirming its predominance in feline populations. Notably, the FeAstV-24 strain isolated in this study clustered with Chinese strains MH253864, MH253866 within *Mamastrovirus* lineage comprising the large Chinese FeAstV strains, suggesting its representation of prevalent FeAstV lineages circulating in China. FeAstV capsid protein is divided into three regions: a conserved N-terminal (amino acid 1–420), a variable central region (aa 421–719) and a conserved C-terminal (aa 720–816). This tripartite architecture mirrors observations in human astroviruses (HAstV) and canine astroviruses (CaAstV) (27, 28). We further found that the variable central region is highly conservative within group 1 or group 2, but significant varied inter groups. Intriguingly, two Chinese FeAstV strains from Shanghai (21SH050-20 and 21SH0505-2) clustered phylogenetically with the recombinant strain FeAstV AH-1-2020, previously reported to harbor an ORF2 segment of porcine astrovirus origin (22). However, definitive evidence of recombination in these Shanghai strains remains elusive due to incomplete genome sequences. Furthermore, phylogenetic proximity between FeAstV and those isolated from bats, foxes, and canines (19, 29) underscores the potential for cross-species transmission and genetic reassortment. Moreover, FeAstV has been reported in children with diarrhea, suggesting the potential possibility of FeAstV infecting humans (3). Collectively, these findings highlight the genomic complexity of FeAstV populations in China and globally, presenting significant challenges for developing targeted prevention strategies against these genetically diverse pathogens.

Isolation of individual FeAstV viruses in cell cultures from such clinical specimens may present a challenge, because of the existence of multiple-virus coinfections (24). In the present study, viral isolation was attempted using F81 cells with the sole single-virus infected fecal sample identified (FeAstV-positive only). Progressive cytopathic effects (CPE), characterized by cell rounding, detachment, and lysis, were observed across passages 1–15, confirming successful isolation of the PAstV5-AH29-2014 strain. The addition of trypsin is important for AstVs isolation in cell cultures, because the capsid proteins of mature astrovirus virions were digested into fragments by trypsin, which is critical for viral entry to initiate infection (30–32). The AstVs from different species such as humans (10, 33–35), calves (36), dogs (5), and pigs (PAstV1) (37, 38) have been successfully isolated by the inclusion of trypsin in cells cultures. To our knowledge, there are no studies reporting the isolation of recently popular FeAstV strains in culture. In this study, we first isolated a FeAstV in F81 cells without using trypsin, the CPE could be observed when the virus is first inoculated into cells. It has reported that porcine astrovirus (PAstV) has been successfully isolated in cell culture without trypsin supplementation (39). Potential mechanisms include: either the virus does not require trypsin-mediated cleavage of the capsid protein for infection, or it utilizes endogenous host proteases/virus-encoded proteases to cleave the capsid protein potentially enabled by viral genetic mutations.

Generally, AstVs are enteroviruses, causing diarrhea in several mammalian species. In particular, HAstVs have been recognized as the second most common cause of viral diarrhea in young children (40). In recent years, FeAstVs have often been detected in both symptomatic and asymptomatic domestic cats, their direct etiological role in feline diarrhea has remained unconfirmed (20, 29, 41, 42). In this study, the oral inoculation route mimics the natural pathway of viral infection (AstV is primarily transmitted via the fecal-oral route). However, the potential impact of digestive tract barriers, such as gastric acid and digestive enzymes on the virus remains unclear. Therefore, subcutaneous injection was combined to ensure rapid and quantifiable viral entry into the bloodstream, bypassing potential physicochemical barriers in the digestive tract (e.g., stomach acid and enzymes). All four inoculated cats exhibited seroconversion and viral shedding, with one developing self-limiting diarrhea lasting 6 days in the absence of co-infections. This confirms FeAstV’s pathogenic potential while highlighting the asymptomatic nature of most infections, aligning with observations in other species where astroviral infections often remain subclinical (43–45). Notably, AstVs demonstrate broad tissue tropism beyond the gastrointestinal tract, having been implicated in neurological disorders in swine (46), avian interstitial nephritis, and duck hepatitis (8). While our study focused on enteric manifestations, the spontaneous resolution of diarrhea precluded histopathological analysis or extraintestinal viral detection. Further investigations incorporating timed necropsies are warranted to explore potential systemic involvement of FeAstV.

The synergistic enhancement of viral pathogenicity in mixed viral infections have been documented in several studies. Co-infection by canine parvovirus (CPV) has been hypothesized to trigger the pathogenicity of coronavirus in dogs (47). The coinfections of classical swine fever virus (CSFV) and PAstV5 strongly suggest that CSFV infection enhances PAstV5 replication through inhibition of IFN-expression (39). Similarly, FPV, an immunosuppressive agent causing severe leukopenia, enteric lesions, and neurological signs (48), frequently co-infected withFeAstV in clinical cases (Table 2) (14). A dual infection by FeAstV and FPV was often diagnosed in a cat presenting with severe gastroenteritis (24). In this study, the F81 cells were co-infected with FPV and FeAstV, showing that FPV increased the replication of FeAstV. A possible explanation that FPV significantly inhibits the production of type I IFN (39), which promots the replication of FeAstV during co-infection. We have confirmed that FeAstV infection has a certain degree of pathogenicity in this study, thus FeAstV infection further contributed to the pathogenicity in mixed infections of FeAstV with FPV. The mixed infections of FeAstV with various viruses should be considered in the diagnosis and treatment of gastroenteric diseases of cats.

A FeAstV strain belonging to the predominant Manstrovirus-2, was successfully isolated from clinical specimens. Experimental infection of cats with this strain recapitulated mild diarrheal disease, with virological monitoring confirming active viral replication and fecal shedding. This work establishes the first direct causative link between FeAstV infection and feline enteric illness, providing critical evidence for its pathogenic role in diarrheal disorders of domestic cats.

## Data Availability

The original contributions presented in the study are included in the article/supplementary material, further inquiries can be directed to the corresponding authors.

## Footnotes

^1^ https://talk.ictvonline.org/taxonomy/
